# Subclinical alterations of resting state functional brain network for adjunctive bright light therapy in nonseasonal major depressive disorder: A double blind randomized controlled trial

**DOI:** 10.3389/fneur.2022.979500

**Published:** 2022-11-09

**Authors:** Chun-Chao Huang, Hui-Chun Huang, Chen-Ju Lin, Chien-Chi Hsu, Chau-Shoun Lee, Yu-Hsin Hsu, Ting-Lin Chen, Wei-Hsin Liao, Yun-Hsiang Wu, Fan-Pei Gloria Yang, Shen-Ing Liu

**Affiliations:** ^1^Department of Radiology, MacKay Memorial Hospital, Taipei, Taiwan; ^2^Department of Medicine, MacKay Medical College, New Taipei, Taiwan; ^3^MacKay Junior College of Medicine, Nursing and Management, Taipei, Taiwan; ^4^Department of Medical Research, MacKay Memorial Hospital, Taipei, Taiwan; ^5^Department of Psychiatry, MacKay Memorial Hospital, Taipei, Taiwan; ^6^Institute of Health and Welfare Policy, National Yang Ming Chiao Tung University, Taipei, Taiwan; ^7^Department of Counseling and Clinical Psychology, Teachers College, Columbia University, New York, NY, United States; ^8^Department of Foreign Languages and Literature, National Tsing Hua University, Hsinchu, Taiwan; ^9^Center for Cognition and Mind Sciences, National Tsing Hua University, Hsinchu, Taiwan; ^10^Department of Radiology, Graduate School of Dentistry, Osaka University, Suita, Japan

**Keywords:** bright light therapy, major depressive disorder, functional MRI, functional network, graph theory

## Abstract

**Introduction:**

The treatment effect of bright light therapy (BLT) on major depressive disorder (MDD) has been proven, but the underlying mechanism remains unclear. Neuroimaging biomarkers regarding disease alterations in MDD and treatment response are rarely focused on BLT. This study aimed to identify the modulatory mechanism of BLT in MDD using resting-state functional magnetic resonance imaging (rfMRI).

**Materials and methods:**

This double-blind, randomized controlled clinical trial included a dim red light (dRL) control group and a BLT experimental group. All participants received light therapy for 30 min every morning for 4 weeks. The assessment of the Hamilton Depression Rating Scale-24 (HAMD-24) and brain MRI exam were performed at the baseline and the 4-week endpoint. The four networks in interest, including the default mode network (DMN), frontoparietal network (FPN), salience network (SN), and sensorimotor network (SMN), were analyzed. Between-group differences of the change in these four networks were evaluated.

**Results:**

There were 22 and 21 participants in the BLT and dRL groups, respectively. Age, sex, years of education, baseline severity, and improvement in depressive symptoms were not significantly different between the two groups. The baseline rfMRI data did not show any significant functional connectivity differences within the DMN, FPN, SN, and SMN between the two groups. Compared with the dRL group, the BTL group showed significantly increased functional connectivity after treatment within the DMN, FPN, SN, and SMN. Graph analysis of the BLT group demonstrated an enhancement of betweenness centrality and global efficiency.

**Conclusion:**

BLT can enhance intra-network functional connectivity in the DMN, FPN, SN, and SMN for MDD patients. Furthermore, BLT improves the information processing of the whole brain.

**Clinical trial registration:**

The ClinicalTrials.gov identifier was NCT03941301.

## Introduction

Major depressive disorder (MDD) is characterized by a depressed mood lasting more than 2 weeks and resulting in emotional distress, functional impairment, health problems, suicide, etc. Worldwide, MDD is currently the leading cause of functional disability. Although antidepressants are effective in treating MDD, the response rate to initial administration is ~50%. A new treatment is recommended if the initial treatment is ineffective after 4–8 weeks (1). One adjunctive treatment is bright light therapy (BLT), which has proven to be a promising treatment for both seasonal and non-seasonal MDDs and is characterized by rapid effects, minor side effects, and low cost (2). The combination of BLT and psychopharmacological therapy is sometimes a better treatment choice for patients with MDD (3). The mechanism of BLT is still unclear but might be different from that of pharmacological agents. One possible mechanism of BLT is thought to be through retinal circuitry, followed by retinofugal projections into the brain, further modulating the functions of mood and cognition. Multiple brain emotional regions can be stimulated by BLT through retinofugal projections from the retina. A key activated region is the dorsal raphe nucleus, which is the main area producing serotonin in the forebrain, with multiple serotonergic axons to the cortex, hippocampus, amygdala, bed nucleus of the stria terminalis, and hypothalamus. The serotonin system is an important neural circuit involved in depression. Therefore, the therapeutic effect of BLT on depression might be related to the modulation of the serotonin system through retinofugal projections (2). Another possible mechanism is that the secretion of pineal melatonin is suppressed by light exposure, and therefore, this effect helps to modulate circadian rhythm and sleep, whose dysfunction may be related to mood disorders (4). Furthermore, BLT has an augmentation treatment effect on depression, even with a refractory response to antidepressant monotherapy (5). A previous randomized double-blind trial showed that a combination of BLT and sertraline was able to enhance the response and remission rates from 38.9 and 18.5% to 70.8 and 41.7%, respectively, compared to response with monotherapy of sertraline (6). In general, BLT has been reported to be effective for treating psychiatric symptoms, yet there is still a large proportion of patients with MDD without significant response to the combination therapy. The underlying mechanism of BLT is still not well-elucidated.

An alternative to understanding the mechanism is to explore neural changes using neuroimaging. Neuroimaging biomarkers of disease alterations in MDD and treatment response have been largely surveyed using resting-state functional magnetic resonance imaging (rfMRI). Several large-scale networks have been discovered in rfMRI data, and are useful tools for investigating cognitive diseases. Three core neurocognitive networks, including the default mode network (DMN), frontoparietal network (FPN), and salience network (SN), have been highlighted for their importance in understanding the integrity of higher cognitive functions (7).

In contrast to previous research focusing on serotonin modulation or a combinatorial pharmaceutical approach, the present study aimed to investigate changes in neural networks whose disruptions were associated with psychiatric disorders. The DMN is associated with self-related cognitive activities such as autobiographical, self-monitoring, and social functions, and is deactivated during stimulus-driven cognitive processing. Impairment of intrinsic functional connectivity of the DMN has been detected in many psychiatric disorders, including MDD (7–9). The FPN, also known as the central executive network, is related to high-level cognitive functions, including planning, decision-making, and the control of attention and working memory. The disruption of the FPN has been identified in various psychiatric disorders, including MDD (7, 8). The SN is involved in the detection and orientation of salient external stimuli and internal events. Abnormal changes in the SN, especially at the anterior insula (AI) node, are consistently implicated in anxiety disorders, which are a common comorbid feature of a variety of psychiatric disorders including MDD (7). In addition, the sensorimotor network (SMN) is composed of motor and sensory areas and is activated during sensorimotor function (10). Functional connectivity alterations of the SMN are also found in patients with MDD compared to those in healthy participants (11). Furthermore, rfMRI can also reflects treatment responses in MDD. For example, low functional connectivity within the cognitive control network in pre-treatment MDD is related to non-remission to escitalopram later in life (12). Reduced baseline functional connectivity within the DMN of the orbitofrontal component can be used to predict clinical responses to duloxetine (1, 13). The treatment responses of patients with MDD include distinct functional deficits. The refractory response shows disrupted functional connectivity, mainly in the thalamocortical circuits. In contrast, the non-refractory response presents a more distributed decreased connectivity in the limbic–striatal–pallidothalamic circuit (14). Additional to pharmaceutical treatments, treatment effects on networks are also found in other types of treatments, such as psychotherapeutic interventions on fronto-limbic networks, electroconvulsive therapy, and repetitive transcranial magnetic stimulation of the subgenual anterior cingulate cortex, DMN, FPN, and dorsolateral prefrontal cortex (8). Despite the aforementioned findings in the altered networks, network connectivity changes resulting from BLT in MDD have not been studied.

The present study hypothesizes that the modulation of disruptions in the brain with MDD should be network-based, as functional impairments associated with the disease may be distributed to disconnectivity in several networks. To evaluate the change of the four specific networks, DMN, FPN, SN, and SMN, the regions of interest (ROIs) of these four networks are pre-defined and then ROI-to-ROI connectivity (RRC) approach and graph analysis are conducted in this study. It is anticipated that the RRC and graph metrics quantifying the central role and global interconnection of ROIs could reveal useful information regarding changes in information flow and integrity in response to BLT.

## Materials and methods

This study was designed as a double-blind randomized controlled clinical trial conducted in the Taipei and Tamsui branches of the MacKay Memorial Hospital from June 2019 to April 2020. The Institutional Review Board of MacKay Memorial Hospital approved the study protocol (IRB number:18MMHIS114e). The ClinicalTrials.gov identifier was NCT03941301. All participants provided written informed consent after the study procedure was fully explained.

### Participant enrollment

The participants were recruited from psychiatrists' outpatient clinics in the two branches of the hospital. The inclusion criteria were as follows: (1) The age of the participants was at least 20 years, (2) Participants had been diagnosed with MDD for at least 6 weeks. The diagnosis of MDD was diagnosed by board-certified psychiatrists using the criteria set by the Diagnostic and Statistical Manual of Mental Disorders, Fifth Edition (DSM-5). The severity of MDD symptoms must be >12 scores on the Hamilton Depression Rating Scale-24 (HAMD-24), (3) Eligible patients had received antidepressants and hypnotics at stable dosages for at least 4 weeks, and with at least one type of antidepressant within therapeutic doses. During this trial, participants and referring psychiatrists were asked not to change their medication regimens. The type of antidepressants used was not limited and the presence of comorbid anxiety disorders was allowed. Participants were excluded if they had any of the following conditions: manic disorder, hypomania, mixed episode, seasonal affective disorder, psychotic disorder, any substance use disorder in the past 30 days, intellectual disability, dementia, cognitive impairment, organic brain syndrome, retinal diseases, severe physical diseases (e.g., cancer), treatment with photosensitizing drugs (St John's Wort), photosensitive epilepsy, or migraine. Psychiatrists and trained research assistants assessed the participants.

### Study protocol

#### Baseline evaluation

For the participants fulfilling the eligibility criteria, research assistants recorded their age, sex, marital status, the highest level of education, years of education, employment status, socioeconomic status, age at onset, number of depressive episodes, anti-depressive agents and hypnotic medications with dosages, and time of the month of enrollment. The HAMD-24 scores were also recorded. The participants were randomly assigned in a 1:1 ratio to the experimental or control group with a block size of six. The researchers performing clinical ratings were blinded to the results of randomization and worked independently from the non-blinded researchers dispensing and instructing the participants on how to use lightboxes.

#### Intervention and follow-up

The participants received one of the two different lightboxes based on the assigned group. All lightboxes looked identical when turned off. Each subject in the experimental group had a 10,000-lux white light unit, which was the commercial 6.5 “W × 8.5” H × 4.5” D in. HAPPYLIGHT^®^ LUCENT™ VT22 (Verilux Company), gives 10,000 lux of natural and ultraviolet-free full-spectrum light. In contrast, each subject in the control group had a 70-lux red light unit, which was custom-made with an appearance identical to that of the experimental white light unit. Participants were provided with standardized verbal and written instructions on the use of the lightbox, including optimal placement of the unit on its desk stand 40–75 cm from the eyes, receiving a 30-min session of light therapy after being awake in the morning every day for 4 weeks, and recording the exposure time to the working lightbox on a standard self-report form. Participants had no knowledge of the different light colors and the color which showed treatment effects. After beginning the light therapy, the researchers assessed the participants at 1, 2, and 4 weeks to monitor compliance and side effects.

### MRI scan and image preprocessing

#### Image acquisition

MRI scanning was performed at baseline and at the 4-week end of the study for all participants. The MRI scans were acquired on two identical Siemens 1.5 T whole-body MRI scanners (Siemens Magnetom Aera, Erlangen, Germany) using a twelve-channel head coil in both the branches of the hospital. Fixation pads were used to reduce bulk head motion. All the acquired images were aligned with the anterior and posterior commissures. Whole-brain resting-state blood oxygen level-dependent (BOLD) rfMRI images were collected using a T2^*^-weighted gradient-echo-planar imaging (EPI) sequence with the following imaging parameters: repetition time (TR)/echo time (TE) = 3,000/40 ms, flip angle = 90°, matrix size = 64 × 64 mm^2^, field of view = 192 × 192 mm^2^, voxel size = 3 × 3 × 3 mm^3^, 42 interleaved axial slices without intersection gap, and 160 continuous image volumes. Further, anatomical three-dimensional T1-weighted images were collected using magnetization-prepared rapid gradient-echo (MPRAGE) sequence with the following imaging parameters: TR/TE/ inversion time (TI) = 2,000/4.7/860 ms, flip angle = 9°, number of excitations (NEX) = 1, field of view = 230 × 230 mm^2^, matrix size = 256 × 256 mm^2^, and voxel size = 0.9 × 0.9 × 0.9 mm^3^. An additional axial T2-weighted fluid-attenuated inversion recovery sequence (2D-T2-FLAIR; TR/TE/TI = 8,000/106/2,371.7 ms, flip angle = 150°, 25 slices, NEX = 1, echo train length = 21, matrix size = 256 × 205 mm^2^, field of view = 230 × 230 mm^2^, slice thickness = 5.0 mm, voxel size = 0.9 × 0.9 × 5.0 mm^3^) was performed. All structural MRI scans were visually reviewed by an experienced neuroradiologist to confirm that the participants were free from any morphological abnormality of the brain.

#### Preprocessing of the rfMRI data

The CONN functional connectivity toolbox (version 17. f), in conjunction with Statistical Parametric Mapping version 12 (SPM12), was used to perform all preprocessing steps (by selecting the default preprocessing pipeline of the CONN) and all subsequent statistical analyses. In the preprocessing pipeline, raw functional images were slice-time corrected, realigned (motion-corrected), unwarped, and co-registered to each subject's MPRAGE image by standard algorithms. Images were then normalized to the Montreal Neurological Institute (MNI) coordinate space, spatially smoothed (8 mm full width at half maximum), and resliced to 2 × 2 × 2 mm^3^ voxels. The ROIs in the present study were derived from the ROI atlas provided by the toolbox (15). The 32 ROIs within this atlas belong to eight networks: the DMN (posterior cingulate cortex, bilateral lateral parietal cortices, medial prefrontal cortex), SMN (bilateral lateral regions and superior region in the sensorimotor network), visual network (medial region, bilateral lateral regions, and occipital region in the visual network), SN (bilateral lateral rostral prefrontal cortices, anterior cingulate cortex, bilateral supramarginal gyri, bilateral anterior insulae), dorsal attention network (bilateral frontal eye fields, bilateral intraparietal sulci), FPN (bilateral posterior parietal cortices, bilateral lateral prefrontal cortices), language network (bilateral inferior frontal gyri, bilateral posterior superior temporal gyri), and cerebellar network (anterior and posterior regions in the cerebellar network). We treated all ROIs as “nodes” within a whole-brain network and focused on the DMN, FPN, SN, and SMN in isolation.

### Concept of image analysis: RRC approach and graph analysis

As MDD results in various functional disabilities with symptoms affecting each other, the underlying disruptions in the brain could be attributed to networks associated with such functional impairments instead of one region or only a few disconnected regions that could not independently cause multiple dysfunctions. Following this view, the investigation of neural underpinnings of improvements in MDD patients should be a network-based analysis that considers entire networks of connections as well as pre-defined ROIs. This RRC approach is in contrast to the whole-brain connectivity approach, where a seed or pre-defined ROI is studied in terms of its connectivity with the rest of the brain (seed-based connectivity). We were more interested in the changes in the level of functional connectivity between each pair of ROIs in the selected networks (DMN, FPN, SN, and SMN) than the connectivity between random seed regions.

Relative to inter-network connectivity, the connectivity within the networks of interest (intra-network connectivity) will provide more information on the disconnected pattern among local network regions and changes in disconnectivity associated with treatment or therapy in patients. Such information will shed light on the neuromodulatory effect of BLT on targeted improvement in MDD, under the assumption that improvement is achieved by connected network ROIs.

To understand neuromodulation at the level of both individual ROIs and connections in the network, in addition to examining the RRC within the selected networks, the present study performed ROI-level graph measures, which were also based on RRC correlations. Graph measures defined non-directional graphs with nodes which were ROIs and edges (suprathreshold connections). A graph adjacency matrix for each subject was computed by thresholding the associated ROI-to-ROI correlation matrix by an absolute or relative threshold. Among the measures computed based on the topological properties of nodes and graphs, we referred to those that reflected the centrality of ROIs and the interconnectedness of the entire network.

Nodes that are central in network organization play an important role in mediating several network connections. The central role can be quantified using measures known as centrality metrics (16). Centrality metrics, such as degree centrality and betweenness centrality, characterize nodes that are likely to influence the behavior of the network and are in the mainstream of information flow (17). The degree of centrality refers to the number of edges connected to a node. Although the degree often proves to identify critical network nodes (18), a node with a higher number of connections in the brain network may not necessarily have ubiquitous connections to other nodes in the network (17).

Betweenness centrality represents the proportion of times a node is part of the shortest path between any two pairs of nodes within a graph (19). By considering nodes along the shortest geodesic paths to be the most central in the network, nodes with high betweenness centrality are believed to be strategically located in the middle zone between several pairs of ROIs and therefore control the flow and integrity of information among nodes in the network. Taken together, the investigation of changes in the centrality of ROIs after therapy in MDD would reveal how the information flow controlled by central nodes is modulated by the BLT.

The global efficiency at a node is defined as an average of the inverse distances between the node and all the other nodes in the same graph. It identifies the degree of global connectedness of each ROI in a graph. Studying the change in global efficiency of ROIs in the DMN, FPN, SN, and SMN after BLT might help us understand the modulated interconnectedness status of each ROI, which consequently changes the information flow and integrity in a network.

### Statistical analysis

Descriptive statistics were used to evaluate the demographic data. To compare between-group differences, the *t*-test was used for continuous variables and the chi-square test for binary variables. Hierarchical linear mixed modeling was performed for the effects of treatment, time, and treatment-by-time interactions.

Image anlysis of rfMRI data was performed after preprocessing. In the second-level analysis, two independent sample *t*-test analysis was conducted to compare the pre-treatment difference between the experimental and control groups and to evaluate the between-group difference in changes after treatments in these two groups using the CONN toolbox. Furthermore, the depression scale (HAMD-24) at baseline and post-treatment of each subject in these two groups was set as a covariate to reanalyze the between-group difference in changes after treatments in these two groups using the CONN toolbox. This analysis was able to reveal the effect of different light therapy on the functional connectivity after considering linear mixed effects of depression.

For graph analysis, we used the automated analysis algorithms of the CONN toolbox based on graph theory to examine the following graph-theoretical metrics:

- The betweenness centrality calculates the number of shortest paths relative to a specific node in a network. The higher the value of a node is, the more likely is to become an important hub.
- Degree centrality is defined for each node as the number of edges from/to each node in the network.
- Global efficiency is an inverse of the mean path length values, representing the information exchange across a pre-defined network of ROIs. High values imply a high functional integration (20).

All statistical tests were two-sided, with a significance threshold of 0.05. The false discovery rate (FDR) was used for multiple comparison when necessary. Data were analyzed using IBM SPSS (version 24.0; IBM, Armonk, New York, USA).

## Results

### Demographic data

A total of 198 participants were initially enrolled in the assessment of eligibility for this study; 155 participants were excluded for the following reasons: 75 participants rejected participation, 58 had a score of HAMD-24 <13, 15 reported a change of the antidepressant, poor compliance on medication or not receiving antidepressants, five presented with ineligible diseases, one was younger than 20 years and one without specific reason recorded. The remaining 43 participants were randomized into the BLT experimental group and the dim red light (dRL) control group. There were 22 and 21 participants in the BLT and dRL groups, respectively. The mean and standard deviation of the age was 47.09 ± 15.03 years old in the BLT group and 42.76 ± 14.30 years old in the dRL group with a *p*-value of 0.34. There were 18 women in the BLT group and 17 women in the dRL group, with a *p*-value of 1.00. The education years were 13.27 ± 4.21 in the BLT group and 14.19 ± 3.53 in the dRL group with a *p*-value of 0.44. Right-handed participants were dominant, and the percentage of right-handed participants was similar in both groups (95.45 vs. 90.48%, *p* = 0.52). The prevalence of anxiety disorders in the BLT group was higher than that in the dRL group, but the difference was not significant (45.45 vs. 23.81%, *p* = 0.14). The mean and standard deviation of the baseline scores of HAMD-24 were 26.32 ± 5.96 for the BLT group and 24.90 ± 8.48 for the dRL group with a *p*-value of 0.52. The scores at the end of this study were 17.50 ± 8.52 for the BLT group and 17.19 ± 10.04 for the dRL group with a *p*-value of 0.91. The differences in the scores from the baseline to the end of this study were 8.82 ± 6.05 for the BLT group and 7.71 ± 10.62 for the dRL group (*p*-value = 0.68; Table 1). Using the hierarchical linear model to compare the effect on depressive symptoms, the BLT group displayed a trend of greater decline in HAMD-24 scores over the study period than in the dRL group but the result was not statistically significant (B = 4.15, *p* = 0.078; Table 2).

**Table 1:** Demographic data for the control and experimental groups.

**Group (case number)**	**BLT (*N* = 22)**	**dRL (*N* = 21)**		
**Variable**	**Mean** ±**SD or No. (%)**		***t*** **/** ***X** ^2^*	***p*** **-value**
Age	47.09 ± 15.03	42.76 ± 14.30	−0.97	*0.34*
Female sex	18 (81.82%)	17 (80.95%)	0.01	*1.00*
Education years	13.27 ± 4.21	14.19 ± 3.53	0.77	*0.44*
Right Hand	21 (95.45%)	19 (90.48%)	0.41	*0.52*
Anxiety disorders	10 (45.45%)	5 (23.81)	2.22	*0.14*
**HAMD-24**
Baseline	26.32 ± 5.96	24.90 ± 8.48	−0.64	*0.52*
End	17.50 ± 8.52	17.19 ± 10.04	−0.11	*0.91*
Difference	8.82 ± 6.05	7.71 ± 10.62	−0.42	*0.68*

BLT, bright light therapy; dRL, dim red light; SD, standard deviation; HAMD, Hamilton Depression Rating Scale. *t*/*X*^2^ means the results of *t* test or Chi-square test.

**Table 2:** Comparison of the effects on bright light therapy and dim red light groups using a hierarchical linear model.

	**Treatment effect**			**Time effect**			**Treatment** × **Time effect**		
	**B**	**95% CI**	***P***	**B**	**95% CI**	***P***	**B**	**95% CI**	***P***
HAMD-24	4.15	−0.48 to 8.78	0.078	−0.90	−2.99 to 1.18	0.391	−1.37	−4.28 to 1.55	0.354

HAMD, Hamilton Depression Rating Scale.

### MRI results

There was no significant difference in functional connectivity within the four evaluated functional networks, including the DMN, FPN, SN, and SMN, in the pre-treatment baseline status between the dRL and BLT groups.

**Figure 1:**
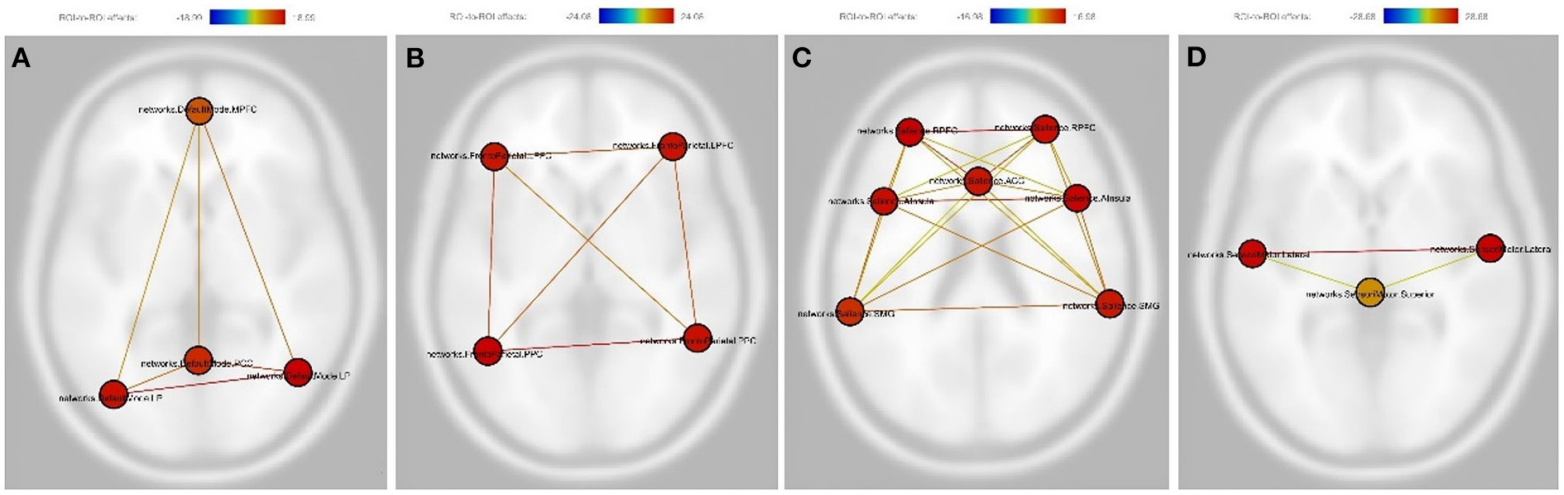
As compared with the dim red light group, the change after treatment in the bright light therapy experimental group showed significantly increased functional connectivity between each node within the default mode network **(A)**, the frontoparietal network **(B)**, the salience network **(C)**, and the sensorimotor network **(D)**. Nodes in **(A)**: PCC, Posterior cingulate cortex; LP (L), left lateral parietal cortex; LP (R), The right lateral parietal cortex; MPFC, Medial prefrontal cortex. Nodes in **(B)**: PPC (L), The left posterior parietal cortex; PPC (R), The right posterior parietal cortex; LPFC (L), The left lateral prefrontal cortex; LPFC (R), The right lateral prefrontal cortex. Nodes in **(C)**: RPFC (L), The left lateral rostral prefrontal cortex; RPFC (R), The right lateral rostral prefrontal cortex; ACC, Anterior cingulate cortex; SMG (L), The left supramarginal gyrus; SMG (R), The right supramarginal gyrus; AInsula (L), The left anterior insula; AInsula (R), The right anterior insula. Nodes in **(D)**: lateral (L), the left lateral region in the sensorimotor network; lateral (R), the right lateral region in the sensorimotor network; Superior, The superior region in the sensorimotor network. The color bar represents the effective connectivity between each node (edge color) and the color of each node means the sum of the effective connectivity of its significant connections.

Compared to the dRL group, the changes after treatment showed significantly increased functional connectivity between each node within the DMN, including the right lateral parietal cortex, left lateral parietal cortex, posterior cingulate cortex, and medial prefrontal cortex (Figure 1A). Similar results were found for the FPN, including the left posterior cingulate cortex, right posterior cingulate cortex, left lateral prefrontal cortex, and right lateral prefrontal cortex (Figure 1B). Moreover, the SN showed similar results, including the right lateral rostral prefrontal cortex, left lateral rostral prefrontal cortex, anterior cingulate cortex, right supramarginal gyrus, right anterior insula, left supramarginal gyrus, and left anterior insula except for the functional connectivity between the left supramarginal gyrus and anterior cingulate cortex (Figure 1C). The SMN displayed similar results, including the left lateral region, a superior region, and the right lateral region, in the sensorimotor network (Figure 1D). The details of the statistical information of these four networks were listed in Supplementary Table S1.

**Figure 2:**
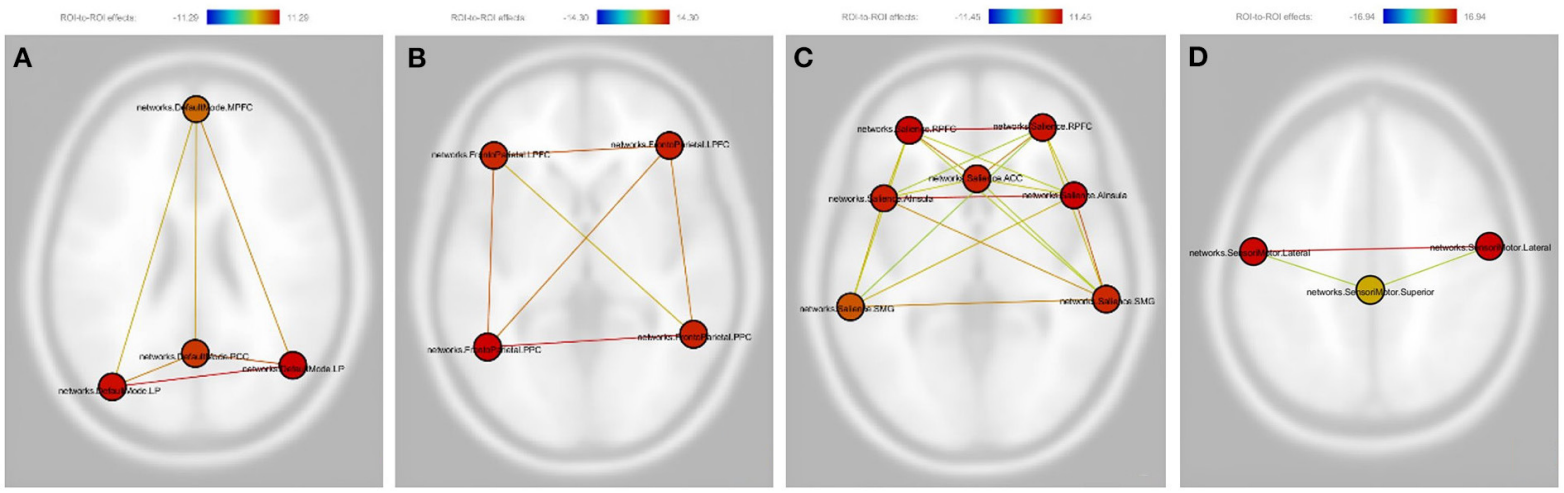
Pictorial demonstration of the results of linear mixed effects of depression in post vs. pre-therapy connectivity. The involved are the following: **(A)** Default mode network: PCC, Posterior cingulate cortex; LP (L), left lateral parietal cortex; LP (R), The right lateral parietal cortex; MPFC, Medial prefrontal cortex. **(B)** Frontoparietal network: PPC (L), The left posterior parietal cortex; PPC (R), The right posterior parietal cortex; LPFC (L), The left lateral prefrontal cortex; LPFC (R), The right lateral prefrontal cortex. **(C)** Salience Network: RPFC (L), The left lateral rostral prefrontal cortex; RPFC (R), The right lateral rostral prefrontal cortex; ACC, Anterior cingulate cortex; SMG (L), The left supramarginal gyrus; SMG (R), The right supramarginal gyrus; AInsula (L), The left anterior insula; AInsula (R), The right anterior insula. **(D)** Sensorimotor network: lateral (L), the left lateral region in the sensorimotor network; lateral (R), the right lateral region in the sensorimotor network; Superior: The superior region in the sensorimotor network. The color bar represents the effective connectivity between each node (edge color) and the color of each node means the sum of the effective connectivity of its significant connections.

Considering linear mixed effects of the depression scale (HAMD-24), the results were the same as above. The BLT group still presented significantly increased functional connectivity between each node within the DMN, FPN, SN (except for the functional connectivity between the left supramarginal gyrus and anterior cingulate cortex) and SMN after treatment as compared with the dRL group (Figure 2). The detailed results were displayed in Supplementary Table S2.

**Figure 3:**
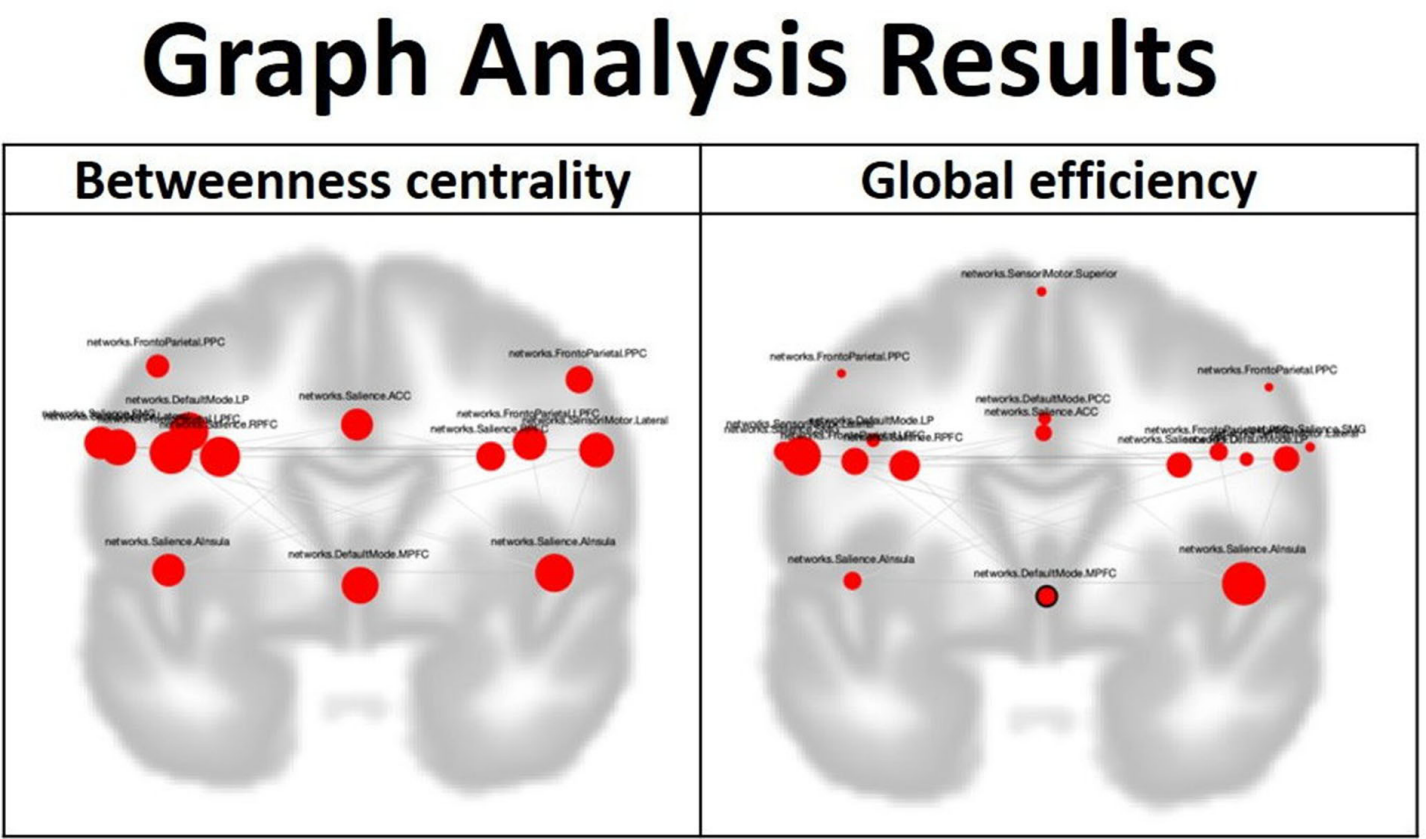
In the graph analysis for the blight light therapy experimental group, after the treatment, betweenness centrality is enhanced over many nodes in the default mode network, frontoparietal network, salience network and sensorimotor network. Furthermore, global efficiency is also elevated between many nodes in these four networks and the four networks as a whole. The involved nodes in the betweenness centrality included the following: Default mode network: LP (L), left lateral parietal cortex; MPFC, medial prefrontal cortex. Salience Network: RPFC (L), The left lateral rostral prefrontal cortex; RPFC (R), The right lateral rostral prefrontal cortex; ACC, Anterior cingulate cortex; SMG (L), The left supramarginal gyrus; AInsula (L), The left anterior insula; AInsula (R), The right anterior insula. Sensorimotor network: lateral (L), left lateral region in the sensorimotor network; lateral (R), right lateral region in the sensorimotor network. Frontoparietal network: PPC (L), The left posterior parietal cortex; PPC (R), The right posterior parietal cortex; LPFC (L), The left lateral prefrontal cortex; LPFC (R), The right lateral prefrontal cortex. The involved nodes in the global efficiency included the following: Default mode network: PCC, posterior cingulate cortex; LP (L), left lateral parietal cortex; MPFC, medial prefrontal cortex; LP (R), right lateral parietal cortex. Salience Network: RPFC (L), The left lateral rostral prefrontal cortex; RPFC (R), The right lateral rostral prefrontal cortex; ACC, Anterior cingulate cortex; SMG (L), The left supramarginal gyrus; SMG (R), The right supramarginal gyrus; AInsula (L), The left anterior insula; AInsula (R), The right anterior insula. Sensorimotor Network: Lateral (L), The left lateral region in sensorimotor network; Lateral (R), The right lateral region in sensorimotor network; Superior, Superior region in sensorimotor network. Frontoparietal network: PPC (L), The left posterior parietal cortex; PPC (R), The right posterior parietal cortex; LPFC (L), The left lateral prefrontal cortex; LPFC (R), The right lateral prefrontal cortex.

In the graph analysis of the dRL group, there was no significant difference between the pre-treatment and post-treatment status. In the graph analysis for the BLT group, there was evidence of multiple nodes in the four functional networks showing significantly increased betweenness centrality compared with the baseline status. There was no significant increase in the degree of centrality in the BLT group. In addition, enhanced global efficiency was found between multiple nodes in the four functional networks and the entire network (Figure 3).

## Discussion

In this study, although there was no significant between-group difference in clinical symptom improvement compared with the control dRL group, the functional connectivity change after treatment showed increased intra-network functional connectivity in the DMN, FPN, SN, and SMN in the BLT group. In the graph analysis results, after BLT, there was evidence of increased betweenness centrality and global efficiency in multiple nodes of these four networks.

The DMN, FPN, and SN are the three major networks highlighted in studies of psychiatric disorders (7). The DMN is deactivated during most stimulus-driven cognitive tasks and is associated with various cognitive functions, such as episodic memory retrieval, autobiographical memory, semantic memory related to internal thought, self-related and social-cognitive processes, value-based decision making, and emotion regulation (7). Abnormal functional connectivity within the DMN has been detected in MDD, especially in the subgenual cingulate, which shows over recruitment in MDD (7, 21). Episodic memory dysfunction in MDD may be related to enhanced connectivity between the subgenual cingulate and ventromedial prefrontal cortex (PFC) and other nodes of the DMN. The symptoms of rumination and recurrent reflective focus on the self, characterized by depression, could be associated with functional connectivity alterations of the PFC nodes in the DMN (7). Although the improvement in clinical symptoms in the BLT group was not significant in this study, the intra-network functional connectivity in the DMN showed a significant increase after treatment in the BLT group as compared with the change in the dRL group. The study of functional connectivity changes in the DMN after the BLT in MDD is sparse in the literature, but one study reported that blue-wavelength light therapy was able to improve structural connectivity between the left lateral parietal cortex and medial PFC in the DMN in patients with mild traumatic brain injury (22). Our findings suggest that the BLT can also improve functional connectivity within the DMN in MDD.

The FPN is a frontoparietal system involved in various cognitively demanding tasks, mainly in actively maintaining and manipulating information in working memory, rule-based problem solving, and decision-making in the context of goal-directed behavior. Disruption of the FPN has been found in MDD, mainly the activation deficits in the dorsolateral PFC and posterior parietal cortex (PPC) (7). Although relatively fewer studies have focused on the functional connectivity change of the FPN in MDD, some inconsistent results have shown hypoconnectivity and hyperconnectivity of the dorsolateral PFC with other brain regions (23). In addition, treatment interventions such as repetitive transcranial magnetic stimulation can modulate the intra-network connectivity of the FPN (24). The results in this study showed increased functional connectivity between each node in the FPN after BLT, which might support the observation that MDD affects not only the activation of nodes in the FPN but also intrinsic functional connectivity. The BLT helps to improve the intrinsic functional connectivity of the FPN and might be able to enhance relevant cognitive functions, such as memory, problem-solving, and decision-making in MDD patients.

The SN is involved in detecting, integrating, and filtering relevant interoceptive, autonomic, and emotional information. In the SN, the AI and dorsal anterior cingulate cortex (ACC) are thought to be part of a functional circuit involved in both attention as well as interoceptive and affective processes (25). In addition, a key function of the SN is to identify the most homeostatically relevant internal and extrapersonal stimuli to guide behavior (7). Hyperactivity of the AI node of the SN has been consistently implicated in anxiety disorders, which are a common comorbid feature of MDD (7, 26). Decreased activation of the insula is associated with symptom reduction in MDD (27). The insula appears to be hyperactive in MDD in response to negative stimuli, and resting-state studies have demonstrated decreased functional connectivity between the insula and the affective brain network (28). The insula mediates the ability to shift attention toward and away from subjective emotional feelings, such as empathy, happiness, love, anger, fear, and sadness, through co-activation with the ACC (29). Because impaired emotion regulation is a crucial feature in MDD and reduced activation of the insula has been linked to the inability to experience emotions, the detected SN connectivity change could also suggest that more persistent emotional dysregulation is related to an insufficient response (25, 28, 30). In this study, increased functional connectivity was found between the bilateral AI and between each AI and the ACC in the SN. The results might suggest that BLT can improve the communication between bilateral AI and between each AI and the ACC, thereby modulating the functions of AI and ACC nodes and improving the ability of emotional processing and anxiety control. The rostral PFC is related to working memory and episodic memory as well as attention to personal or others' emotions and mental states (31). The results of this study also show that the BLT helps to increase functional connectivity between the bilateral rostral PFC and between each rostral PFC and ACC, serving an additional positive effect on the functions of processing memory and emotion in participants with MDD *via* the neuromodulatory effect on the SN.

The SMN, compared with the abovementioned three high cognitive function networks, is a primary function network. It is composed of motor and sensory areas and is activated during sensorimotor functions (10). Furthermore, MDD disrupts sensorimotor processing, and normalization of the SMN could be a predictor of treatment response in MDD (8). Sensorimotor stimulation can also modulate mood and depressive symptoms (32). In this study, after BLT, increased functional connectivity between the bilateral lateral regions of the SMN and between each lateral region and the superior region was observed. A previous study showed that the bilateral connection of the SMN was interrupted after stroke but normalized after rehabilitation (33). The increased functional connectivity between the bilateral lateral regions of the SMN in this study might reflect the treatment effect of BLT on the solidification of the network in MDD. In addition, there is increased functional connectivity between each lateral region and the superior region of the SMN. The superior region of the SMN covers the locations of the supplementary motor area (SMA) and pre-SMA, which manage the execution and planning of motor tasks, respectively. The lateral regions of the SMN are mainly composed of motor and sensory cortices (34). The increased functional connectivity between the lateral and superior regions might suggest better communication from the planning and execution of a motor task to the motor cortex after BLT.

Graph analysis is based on the concept that information processing requires brain-wide integration and that a signal from one brain system is globally broadcast and available to all other relevant brain systems. The analytic method has been applied to study some socio-emotional processes (35). In this analysis, betweenness centrality reflects the node's role in acting as a bridge between separate clusters; a high nodal centrality means the node is an important hub in the network (36). The measurement of betweenness centrality can reflect the importance of a node in controlling the flow and integrity of information within a network. In this study, most nodes in the selected four networks demonstrate elevated betweenness centrality after the BLT. This finding suggests that the effect of BLT can improve the ability of multiple nodes in controlling the flow and integrity of information. Global efficiency is used to represent the efficiency of information transfer across all brain regions (35). The measurement of global efficiency can reflect the global interconnected status of the nodes within a network. All the nodes in these four networks demonstrate elevated global efficiency after BLT, implying that the effect of BLT can enhance the functional connection of nodes and help information flow to be more efficiently exchanged. In addition to the effect of enhancing intra-network functional connectivity of the DMN, FPN, SN, and SMN in MDD patients, based on the results of graph analysis, the BLT also helps global information processing of the brain by improving the flow and integrity of information and the efficiency of global information exchange among the nodes in these four networks.

## Limitations

The number of enrolled participants was limited, and the analytical results might not reflect minor functional alterations. All participants were symptomatic and received antidepressant treatments. Although the medication was not changed during the study period, the types of medication were diverse, which might be a potential bias. In addition, all participants were receiving at least one type of antidepressant, and the BLT might have interacted with the antidepressant treatment. Since antidepressants are the main therapeutic choice with more solid evidence of clinical benefit, the neuromodulatory effect of BLT might be obscured. This study was conducted in a subtropical country with relatively abundant daylight; therefore, the effect of BLT might be suppressed, which is probably the reason why the clinical depressive symptoms did not significantly improve compared with those in the control group.

## Conclusion

We showed that BLT can enhance intra-network functional connectivity in the DMN, FPN, SN, and SMN in patients with MDD. In addition, BLT strengthens the flow and integrity of information and the efficiency of global information exchange among the nodes in these four networks. Although the clinical depressive symptoms do not significantly improve in the BLT group, there is functional imaging evidence of a positive neuromodulatory effect of BLT, and a future study focusing on the improvement of detailed cognitive functional evaluation other than depressive symptoms would be valuable. In addition, the method of how to use BLT in order to enhance the clinical significance in a subtropic location would be an interesting topic to be clarified in future studies.

## Data availability statement

The raw data supporting the conclusions of this article will be made available by the authors, without undue reservation.

## Ethics statement

The studies involving human participants were reviewed and approved by the Institutional Review Board of MacKay Memorial Hospital. The patients/participants provided their written informed consent to participate in this study.

## Author contributions

C-CHu: conceptualization, writing—original draft, writing—review and editing, and funding acquisition. H-CH: methodology, formal analysis, investigation, and data curation. C-JL, C-CHs, and C-SL: resources. Y-HH: investigation and data curation. T-LC and W-HL: investigation. Y-HW: formal analysis. F-PY: supervision, methodology, formal analysis, and writing—review and editing. S-IL: supervision, methodology, resources, and writing—review and editing. All authors contributed to the article and approved the submitted version.

## Funding

This research was co-sponsored by Mackay Memorial Hospital (Grant numbers: MMH 108-147 and MMH-109-98) and Ministry of Science and Technology of Taiwan (Grant number: MOST 108-2314-B-195-001-).

## Conflict of interest

The authors declare that the research was conducted in the absence of any commercial or financial relationships that could be construed as a potential conflict of interest.

## Publisher's note

All claims expressed in this article are solely those of the authors and do not necessarily represent those of their affiliated organizations, or those of the publisher, the editors and the reviewers. Any product that may be evaluated in this article, or claim that may be made by its manufacturer, is not guaranteed or endorsed by the publisher.
