# Supplementary material for: Subclinical alterations of resting state functional brain network for adjunctive bright light therapy in nonseasonal major depressive disorder: A double blind randomized controlled trial
Source: Front Neurol. 2022 Nov 9;13:979500. doi: 10.3389/fneur.2022.979500 (PMC9681819; doi:10.3389/fneur.2022.979500)
Supplement: Supplementary file 1 [file Data_Sheet_1.docx]

Table S1. Between-group functional connectivity change after treatment between each node within the networks.

| **Default Mode Network** | | | | |
| --- | --- | --- | --- | --- |
| ROI source | ROI target | Statistic | p-unc | p-FDR |
| LP (R) | LP (L) | T(41) = 18.99 | <0.001** | <0.001** |
|  | PCC | T(41) = 14.35 | <0.001** | <0.001** |
|  | MPFC | T(41) = 9.98 | <0.001** | <0.001** |
| LP (L) | PCC | T(41) = 10.49 | <0.001** | <0.001** |
|  | MPFC | T(41) = 7.40 | <0.001** | <0.001** |
| PCC | MPFC | T(41) = 8.51 | <0.001** | <0.001** |
| **Frontoparietal Network** | | | | |
| ROI source | ROI target | Statistic | p-unc | p-FDR |
| PPC (L) | PPC (R) | T(41) = 24.08 | <0.001** | <0.001** |
|  | LPFC (L) | T(41) = 17.72 | <0.001** | <0.001** |
|  | LPFC (R) | T(41) = 14.47 | <0.001** | <0.001** |
| PPC (R) | LPFC (R) | T(41) = 15.78 | <0.001** | <0.001** |
|  | LPFC (L) | T(41) = 11.36 | <0.001** | <0.001** |
| LPFC (L) | LPFC (R) | T(41) = 15.88 | <0.001** | <0.001** |
| **Salience Network** | | | | |
| ROI source | ROI target | Statistic | p-unc | p-FDR |
| RPFC (R) | RPFC (L) | T(41) = 16.98 | <0.001** | <0.001** |
|  | ACC | T(41) = 10.09 | <0.001** | <0.001** |
|  | SMG (R) | T(41) = 7.24 | <0.001** | <0.001** |
|  | AInsula (R) | T(41) = 5.87 | <0.001** | <0.001** |
|  | SMG (L) | T(41) = 4.63 | <0.001** | <0.001** |
|  | Alnsula (L) | T(41) = 4.17 | <0.001** | <0.001** |
| RPFC (L) | ACC | T(41) = 12.13 | <0.001** | <0.001** |
|  | Alnsula (L) | T(41) = 7.11 | <0.001** | <0.001** |
|  | SMG (L) | T(41) = 6.27 | <0.001** | <0.001** |
|  | SMG (R) | T(41) = 5.58 | <0.001** | <0.001** |
|  | Alnsula (R) | T(41) = 4.62 | <0.001** | <0.001** |
| Alnsula (R) | Alnsula (L) | T(41) = 15.20 | <0.001** | <0.001** |
|  | SMG (R) | T(41) = 9.49 | <0.001** | <0.001** |
|  | SMG (L) | T(41) = 8.38 | <0.001** | <0.001** |
|  | ACC | T(41) = 7.75 | <0.001** | <0.001** |
| SMG (R) | SMG (L) | T(41) = 10.51 | <0.001** | <0.001** |
|  | Alnsula (L) | T(41) = 8.01 | <0.001** | <0.001** |
|  | ACC | T(41) = 5.13 | <0.001** | <0.001** |
| Alnsula (L) | SMG (L) | T(41) = 9.54 | <0.001** | <0.001** |
|  | ACC | T(41) = 7.02 | <0.001** | <0.001** |
| **Sensorimotor Network** | | | | |
| ROI source | ROI target | Statistic | p-unc | p-FDR |
| Lateral (L) | Lateral (R) | T(41) = 28.68 | <0.001** | <0.001** |
|  | Superior | T(41) = 4.74 | <0.001** | <0.001** |
| Lateral (R) | Superior | T(41) = 5.29 | <0.001** | <0.001** |

**Default Mode Network**:

**: p<0.0001; PCC: Posterior cingulate cortex; LP (L): The left lateral parietal cortex; LP (R): The right lateral parietal cortex; MPFC: Medial prefrontal cortex

**Frontoparietal Network**:

**: p<0.0001; PPC (L): The left posterior parietal cortex; PPC (R): The right posterior parietal cortex; LPFC (L): The left lateral prefrontal cortex; LPFC (R): The right lateral prefrontal cortex

**Salience Network**:

**: p<0.0001; RPFC (L): The left lateral rostral prefrontal cortex; RPFC (R): The right lateral rostral prefrontal cortex; ACC: Anterior cingulate cortex; SMG (L): The left supramarginal gyrus; SMG (R): The right supramarginal gyrus; Alnsula (L): The left anterior insula; Alnsula (R): The right anterior insula

**Sensorimotor Network:**

**: p<0.0001; Lateral (L): The left lateral region in sensorimotor network; Lateral (R): The right lateral region in sensorimotor network; Superior: The superior region in sensorimotor network

Table S2. Detailed results of linear mixed effects of depression in post versus pre-therapy connectivity

| **Default Mode Network** | | | | |
| --- | --- | --- | --- | --- |
| ROI source | ROI target | Statistic | p-unc | p-FDR |
| LP (R) | LP (L) | T(39) = 11.29 | <0.001** | <0.001** |
|  | PCC | T(39) = 8.51 | <0.001** | <0.001** |
|  | MPFC | T(39) = 6.46 | <0.001** | <0.001** |
| LP (L) | PCC | T(39) = 6.81 | <0.001** | <0.001** |
|  | MPFC | T(39) = 5.21 | <0.001** | <0.001** |
| PCC | MPFC | T(39) = 5.44 | <0.001** | <0.001** |
| **Frontoparietal Network** | | | | |
| ROI source | ROI target | Statistic | p-unc | p-FDR |
| PPC (L) | PPC (R) | T(39) = 14.30 | <0.001** | <0.001** |
|  | LPFC (L) | T(39) = 10.37 | <0.001** | <0.001** |
|  | LPFC (R) | T(39) = 9.12 | <0.001** | <0.001** |
| PPC (R) | LPFC (R) | T(39) = 8.94 | <0.001** | <0.001** |
|  | LPFC (L) | T(39) = 6.24 | <0.001** | <0.001** |
| LPFC (L) | LPFC (R) | T(39) = 10.72 | <0.001** | <0.001** |
| **Salience Network** | | | | |
| ROI source | ROI target | Statistic | p-unc | p-FDR |
| RPFC (R) | RPFC (L) | T(39) = 11.45 | <0.001** | <0.001** |
|  | ACC | T(39) = 6.84 | <0.001** | <0.001** |
|  | SMG (R) | T(39) = 4.41 | <0.001** | <0.001** |
|  | AInsula (R) | T(39) = 4.38 | <0.001** | <0.001** |
|  | SMG (L) | T(39) = 2.12 | 0.0403 | 0.0403 |
|  | Alnsula (L) | T(39) = 2.63 | 0.0122 | 0.0122 |
| RPFC (L) | ACC | T(39) = 7.10 | <0.001** | <0.001** |
|  | Alnsula (L) | T(39) = 4.68 | <0.001** | <0.001** |
|  | SMG (L) | T(39) = 3.76 | <0.001** | <0.001** |
|  | SMG (R) | T(39) = 3.24 | 0.0024 | 0.0024 |
|  | Alnsula (R) | T(39) = 3.33 | 0.0019 | 0.0023 |
| Alnsula (R) | Alnsula (L) | T(39) = 10.74 | <0.001** | <0.001** |
|  | SMG (R) | T(39) = 8.17 | <0.001** | <0.001** |
|  | SMG (L) | T(39) = 4.80 | <0.001** | <0.001** |
|  | ACC | T(39) = 4.15 | <0.001** | <0.001** |
| SMG (R) | SMG (L) | T(39) = 6.64 | <0.001** | <0.001** |
|  | Alnsula (L) | T(39) = 6.15 | <0.001** | <0.001** |
|  | ACC | T(39) = 3.00 | 0.0047 | 0.0047 |
| Alnsula (L) | SMG (L) | T(39) = 5.06 | <0.001** | <0.001** |
|  | ACC | T(39) = 3.86 | <0.001** | <0.001** |
| **Sensorimotor Network** | | | | |
| ROI source | ROI target | Statistic | p-unc | p-FDR |
| Lateral (L) | Lateral (R) | T(39) = 16.94 | <0.001** | <0.001** |
|  | Superior | T(39) = 3.70 | <0.001** | <0.001** |
| Lateral (R) | Superior | T(39) = 4.05 | <0.001** | <0.001** |

**Default Mode Network**:

**: p<0.0001; PCC: Posterior cingulate cortex; LP (L): The left lateral parietal cortex; LP (R): The right lateral parietal cortex; MPFC: Medial prefrontal cortex

**Frontoparietal Network**:

**: p<0.0001; PPC (L): The left posterior parietal cortex; PPC (R): The right posterior parietal cortex; LPFC (L): The left lateral prefrontal cortex; LPFC (R): The right lateral prefrontal cortex

**Salience Network**:

**: p<0.0001; RPFC (L): The left lateral rostral prefrontal cortex; RPFC (R): The right lateral rostral prefrontal cortex; ACC: Anterior cingulate cortex; SMG (L): The left supramarginal gyrus; SMG (R): The right supramarginal gyrus; Alnsula (L): The left anterior insula; Alnsula (R): The right anterior insula

**Sensorimotor Network:**

**: p<0.0001; Lateral (L): The left lateral region in sensorimotor network; Lateral (R): The right lateral region in sensorimotor network; Superior: The superior region in sensorimotor network
